# Longer Lifetime of BC from Fossil Fuel Combustion than from Biomass Burning: Δ^14^C Evidence

**DOI:** 10.1021/acs.est.4c10040

**Published:** 2025-02-25

**Authors:** Chaoliu Li, Zhaofu Hu, Shichang Kang, Elena N. Kirillova, Fangping Yan, Pengfei Chen, Guofeng Shen, Thompson T. Jake, Örjan Gustafsson

**Affiliations:** † Key Laboratory of Cryospheric Science and Frozen Soil Engineering, Northwest Institute of Eco-Environment and Resources, Chinese Academy of Sciences, Lanzhou 730000, China; ‡ Department of Environmental Science and the Bolin Centre for Climate Research, Stockholm University, Stockholm 10691, Sweden; § College of Urban and Environmental Sciences, Peking University, Beijing 100871, China; ∥ University of Chinese Academy of Sciences, Beijing 100049, China; ⊥ Department of Earth and Planetary Sciences, Yale University, New Haven, Connecticut 06511, United States; # Institute of Medicine, Ecology and Physical Education, Ulyanovsk State University, Ulyanovsk 432017, Russian Federation

**Keywords:** Carbonaceous Aerosol, Fractionation, δ^14^C, Precipitation, Tibetan Plateau

## Abstract

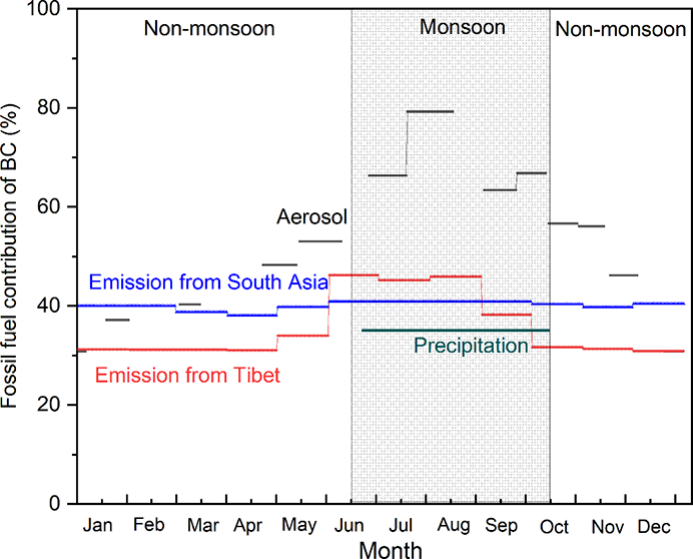

Black carbon (BC) significantly contributes to atmospheric warming and glacier melting. However, the atmospheric lifetime of BC from different fuel sources remains poorly constrained. By analyzing Δ^14^C of BC in PM_2.5_ and precipitation samples collected for three years at a remote site in the Tibetan Plateau, we found that BC from fossil fuel contribution (*f*_fossil_ BC) in PM_2.5_ exhibited greater seasonal variation than those from South Asia and emission inventories. Precipitation-induced fractionation between fossil fuel combustion-derived BC (BC_ff_) and biomass burning-derived BC (BC_bb_) resulted in an increase of *f*_fossil_ BC to 68 ± 7% during the wet monsoon season, which is significantly higher than levels measured at a background site in South Asia and in simultaneously collected precipitation samples. Our findings provide direct evidence that the lifetime of BC_ff_ is longer than that of BC_bb_ during the monsoon season. These results emphasize the increased climate forcing of BC_ff_ relative to BC_bb_ at remote sites receiving long-range transported BC.

## Abstract 2

Our study provides compelling evidence that BC sourced from fossil fuel burning has a longer lifetime than that emitted from biomass combustion, which is important for understanding behavior of BC in the atmosphere.

## 1 Introduction

Black carbon (BC) aerosols are considered one of the most significant atmospheric warming agents due to their strong climate forcing potential.^1^ When deposited on snow and ice, BC reduces surface albedo, which accelerates glacier melt by absorbing sunlight.^2,3^ This process affects the evolution of freshwater downstream from glaciers. However, identifying the sources of atmospheric BC, especially in remote regions, remains challenging due to limited in situ data, hindering efforts to mitigate BC emissions.

This challenge is particularly notable in the Himalayas and the Tibetan Plateau (TP)—one of the most remote regions on Earth, characterized by extreme terrain variations. Numerous studies have shown that BC is a critical driver of glacier retreat in the TP, particularly in the Himalayas, which are directly impacted by substantial BC emissions from heavily polluted South Asia.^3,4^ This makes investigating BC sources in the TP a major research focus.^5−7^ However, there is ongoing debate over the dominant sources of BC in the TP. Some studies suggest that BC from outside the TP is the primary contributor to both atmospheric and glacier-deposited BC in the region.^8^ Others argue that locally sourced BC from within the TP itself also plays an important role.^5,7,9^

Radiocarbon (Δ^14^C) has been shown to be an effective method for distinguishing BC from biomass combustion and fossil fuel combustion sources (*f*_fossil_ BC),^10−13^ especially in remote regions such as the Arctic^14,15^ and the TP.^5,16^ Additionally, studying the behavior of BC during the wet deposition process is an important scientific question related to its lifetime in the atmosphere. Therefore, selecting a representative remote site in the TP with minimal local influence is essential for investigating the sources of BC and the potential factors affecting its deposition.

**Figure 1:**
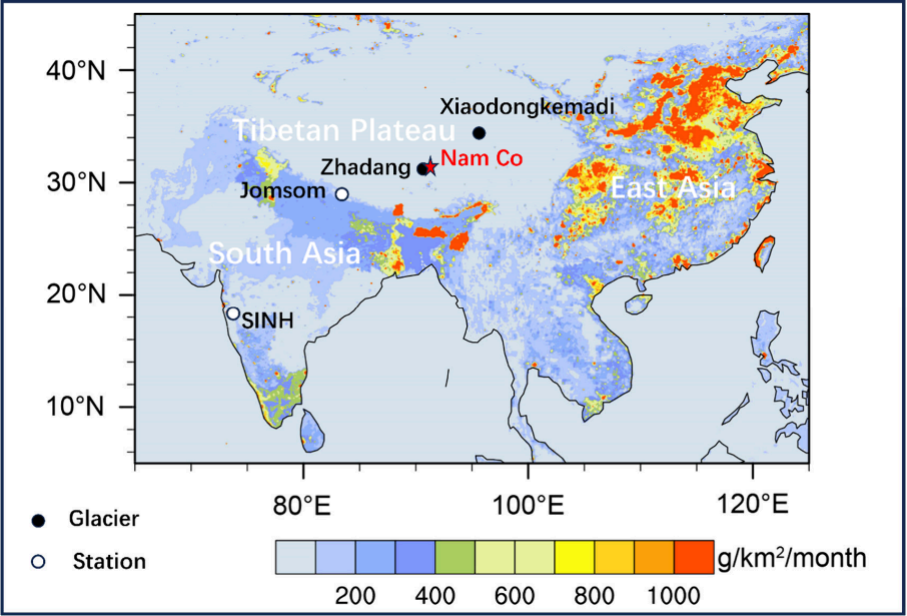
Location of the study site and related sites in South Asia and the Himalayas. The background map shows predicted monthly mean BC emissions of South Asia and East Asia (https://gems.pku.edu.cn).

Nam Co station is considered one of the most typical and representative remote stations in the inner TP^17^ (Figure 1). It has the lowest Aerosol Optical Depth (AOD) in the TP,^18^ and its annual BC concentration is among the lowest across the entire TP.^19,20^ The climate at Nam Co station is characterized by distinct wet monsoon and dry nonmonsoon seasons. Therefore, the clean atmosphere, the reception of BC from long distances, and the clear differentiation between wet and dry seasons make the Nam Co station an ideal site for investigating BC sources.

In addition to BC sources, this study offers a valuable opportunity to explore the fractionation of fossil fuel combustion-derived BC (BC_ff_) and biomass burning sourced BC (BC_bb_) during wet deposition. BC_ff_ is generally considered more hydrophobic and has a smaller particle size than that of BC_bb_, making it more likely to be transported over long distances.^21^ So far, the distinct mixing states of BC from these two fuel types have not been explicitly incorporated into most climate models.^22^ A previous study at Nam Co station provided direct evidence of lower *f*_fossil_ in water-insoluble particulate carbon (WIPC) in precipitation samples compared to aerosols.^23^ A similar phenomenon was also observed in the remote Arctic, where the *f*_fossil_ BC in snow was lower than in aerosols, implying fractionation between BC_ff_ and BC_bb_ during wet deposition.^24^ Therefore, a similar fractionation mechanism should occur at other sites around the world. Proving this will have significant implications for understanding the atmospheric lifetime and different climate forcing of BC from fossil fuel and biomass combustion sources. Thus, the remote Nam Co station provides an ideal site for investigating this mechanism. We observed greater seasonal variations in *f*_fossil_ BC at Nam Co station compared to a background site in South Asia. Additionally, the *f*_fossil_ BC value at Nam Co station during the monsoon season was higher than those of the background site of South Asia and estimations from emission inventories. We propose that this discrepancy is primarily due to fractionation between BC_ff_ and BC_bb_, mainly driven by wet deposition. As a result, precipitation increases the BC_ff_/BC_bb_ ratio in the atmosphere, compared to what is predicted from emission inventories. This phenomenon is particularly pronounced at remote site in the world.

## 2 Materials and Methods

### 2.1 Study Sites

Aerosol and precipitation samples were collected at the Nam Co station from December 2016 to July 2019 (Figure 1). Nam Co station is located in an open area of the inner TP (30°46′23.80″N, 90°57′48.88″E, 4747 m a.s.l.). It is considered one of the most typical and representative remote stations in the inner TP.^17^ Nam Co station experiences two distinct climate regimes: the nonmonsoon season from October to May, dominated by westerly winds, and the monsoon season from June to September, governed by the Indian monsoon system. The nonmonsoon season is typically dry, with occasional dust storms, while the monsoon season brings heavy precipitation from South Asia. Traditionally, local residents migrate from the south to the north bank of Nam Co at the end of September to avoid cold weather and heavy snow, making the Nam Co station a typical remote site during this time. In early April, residents return to the south bank to access better grazing for their yaks and sheep (Figure S1). Because local emissions contribute to the atmosphere at some sites on the TP,^9^ biomass combustion from local residents may influence BC at Nam Co station from April to September. However, the absence of residents on the north bank during this period eliminates local emissions there. Therefore, PM_2.5_ samples were collected simultaneously at both the Nam Co station and the north bank from April to September to assess potential local contributions by comparing Δ^14^C values of BC between these two sites. To investigate the different behaviors of BC_bb_ and BC_ff_ transported from South Asia to the TP, f_fossil_ BC data from total suspended particulates (TSP) samples collected at Jomsom^25^ and Sinhagad Observatory Station (SINH)^26^ were included for comparison with BC composition in the Indian source region (Figure 1). Since f_fossil_ BC exhibits relatively consistent values across different sites in South Asia,^27^ f_fossil_ BC of SINH is expected to be representative of that in the Indo-Gangetic Plain (IGP). The f_fossil_ BC values from Jomsom and SINH were obtained using consistent protocols, ensuring comparability with the data from Nam Co. It is worth noting that f_fossil_ BC values from TSP samples were generally slightly lower than those from the PM_2.5_ samples. Therefore, the f_fossil_ BC values from Jomsom and SINH could be slightly underestimated if they were also based on PM2.5 samples.

### 2.2 PM_2.5_ and Precipitation Sample Collection

To minimize bias in BC measurements due to relatively high mineral dust content in TSP samples at Nam Co station,^19^ we focused on PM_2.5_ samples. A total of 30 PM_2.5_ samples were collected using 90 mm preburned (550 °C, 6 h) quartz fiber filters (Whatman Corp) with an aerosol cyclone equipped with a flow meter that record the volume of air passed through the filters at standard conditions (TH150-A, Wuhan Tianhong INST Group, China) from December 2016 to July 2019. Additionally, six PM_2.5_ samples were collected from June 2018 to September 2019 on the roof of a local resident’s home at the north bank (30°50′04.40″N, 91°06′49.41″E, 4764 m a.s.l.) (Figure S1; Table S1). Due to low BC concentration in the atmosphere, PM_2.5_ samples were collected over extended periods (around 2 weeks), similar to methods used in Arctic studies.^14,28^ Two field blank filters were also collected during each season by exposing the filters in each sampler without pumping. For comparison, precipitation samples were also collected at the Nam Co station. Because of the low BC concentrations in precipitation^29^ and the limited precipitation amount per event, multiple precipitation events over a continuous period were combined to obtain enough BC for Δ^14^C analysis (Table S2). Despite these limitations, the potential fractionation between BC_ff_ and BC_bb_ can still be effectively assessed by treating the monsoon season as a single entity. A total of six filter samples containing BC from precipitation were collected during the monsoon season.

### 2.3 Analytical Methods

The BC mass concentrations in filtered precipitation and PM_2.5_ samples were pretreated and quantified using the methods described in our previous work.^5^ In brief, a 1 cm^2^ punch of each collected sample was exposed to 37% hydrochloric acid (HCl) for 24 h to remove inorganic carbon. Residual acid was then evaporated at 60 °C for 2 h. Elemental carbon (EC, chemically equivalent to BC) concentration was measured by using the NIOSH 5040 protocol. For ^14^C measurements, the required filter area was calculated based on BC concentration and subjected to the same protocol. The CO_2_ produced was cryotrapped for measurement. Δ^14^C of trapped CO_2_ was measured at the Tandem Laboratory in Uppsala University, Sweden.^27^ Due to the inert nature of BC, no significant isotopic fractionation is expected during its long-range transport during the dry, low-precipitation nonmonsoon season.^30,31^ Similar analysis methods have been widely used in previous studies for both aerosol^10−12,31,32^ and snow/ice core samples.^5,33^ The potential impact of organic carbon charring on the estimated Δ^14^C of BC was considered negligible.^12^

### 2.4 BC Concentration and Δ^14^C Calculation

BC concentration in the atmosphere was calculated as
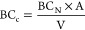
1where BC_C_ is BC concentration in the atmosphere; BC_N_ is BC concentration measured by NIOSH protocol; A is the area of particle loaded on the filter (50.24 cm^2^); V is standard air volume passed through the filter for each sample.

Δ^14^C of BC was calculated as
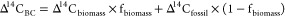
2where Δ^14^C_BC_ is the measured radiocarbon content of BC; Δ^14^C_fossil_ is the fossil fuel combustion end member (−1000‰), and Δ^14^C_biomass_ is the biomass burning end member (+70‰).^5^

## 3 Results and Discussion

### 3.1 f_fossil_ BC in PM_2.5_ and Precipitation Samples

Despite potential influences of local sources (e.g., human activities near Nam Co station), *f*_fossil_ BC in PM_2.5_ samples at Nam Co station were similar to those measured at the north bank, suggesting a minimal impact from local emissions during the study period (Figure S2). Therefore, BC collected at Nam Co station represents a well-mixed sample from both local and remote sources. This suggests that the data obtained at Nam Co station are representative of the remote inner TP, consistent with findings from a previous study.^17^ A similar limited spatial variation in *f*_fossil_ BC is also found in snowpit samples from different glaciers in the inner TP. For instance, *f*_fossil_ BC in snowpit samples from Zhadang glacier and Xiaodongkemadi glacier, located approximately 330 km apart, were 31 ± 1% and 29 ± 10%, respectively^5^ (Figure 1), implying well mixed BC in the atmosphere and consistent *f*_fossil_ values at different sites within the remote inner TP.

**Figure 2:**
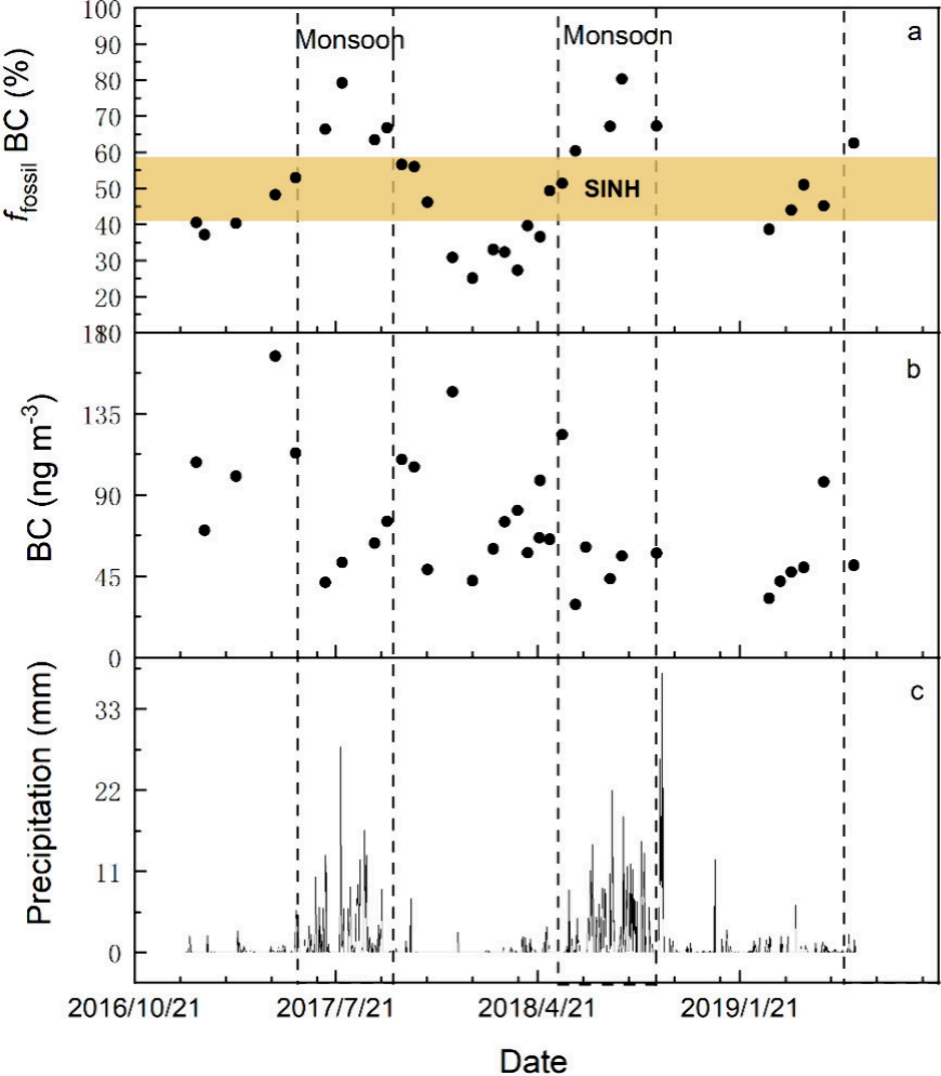
Seasonal variations of *f*_fossil_ BC (a), BC concentration in PM_2.5_ samples (b), and precipitation amounts (c) during the study period at Nam Co Station.

**Figure 3:**
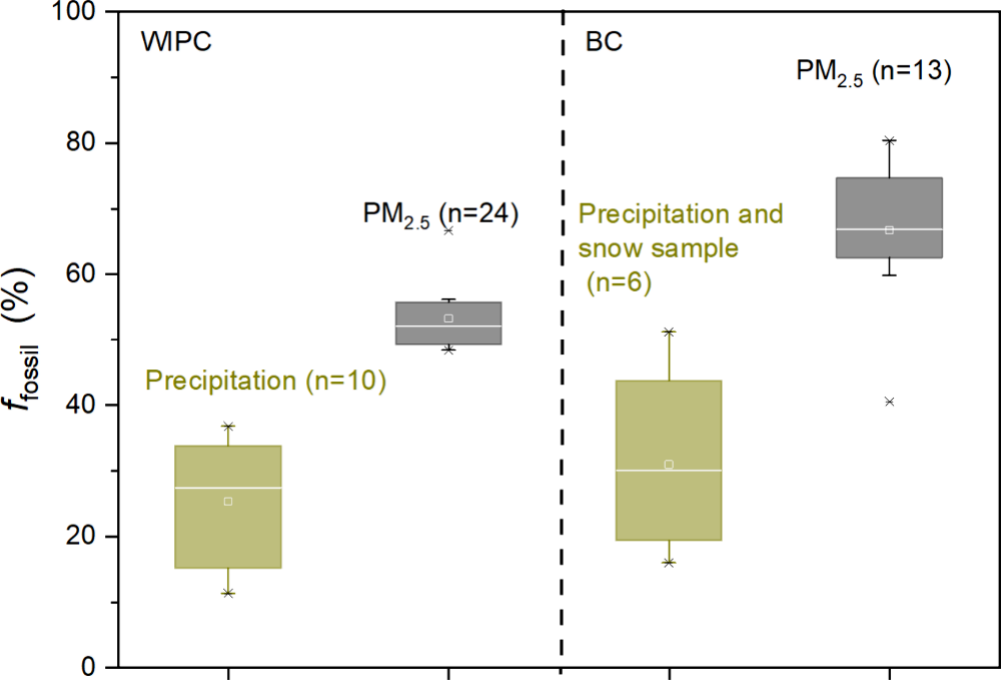
Comparison of *f*_fossil_ BC and *f*_fossil_ WIPC^23^ between precipitation/snow samples and PM_2.5_ samples during the monsoon season at Nam Co station.

The average BC concentration at the study site during the study period was 72 ± 34 ng m^–3^, consistent with previous measurements by an aethalometer^34^ and filter samples at this site.^19^ BC concentrations were generally low during the monsoon season and high during the premonsoon season, similar to earlier findings at this site^34^ (Figure 2b). This may reflect the combined effects of BC source emissions and the deposition rates. For instance, the precipitation amount and BC emissions reached their highest and lowest values, respectively, during monsoon period around Nam Co station (Figure 1c, Table S3). Both factors contribute to the observed low BC concentrations during this period. The average *f*_fossil_ BC in PM_2.5_ samples at Nam Co station was 49 ± 15%, consistent with our previously reported value at the same site (44 ± 6%),^5^ indicating stable BC fuel sources across different years. However, *f*_fossil_ BC in PM_2.5_ samples at the Nam Co station increased to 68 ± 7% during the monsoon season, significantly higher than that in precipitation samples (35 ± 13%) (Figure 3). This difference highlights the preferential removal of BC_bb_ during wet deposition, leaving a higher proportion of BC_ff_ in the atmosphere. A similar phenomenon has been observed at other sites worldwide. For instance, *f*_fossil_ BC in precipitation samples from Dübendorf, Switzerland, was 52 ± 7%,^35^ significantly lower than the 83 ± 10% observed in aerosols in nearby Zürich.^11^ Similarly, the Δ^14^C value of BC in snow samples from spring (−323.2 ± 115.5‰) at the remote site of Alert, Canada was lower than that of aerosol (−581 ± 78.7 ‰) collected simultaneously.^24^ Therefore, the preferential wet removal of BC_bb_ over BC_ff_ is a widespread phenomenon occurring in both remote and urban areas worldwide.

### 3.2 Seasonal Variations of f_fossil_ BC in PM_2.5_ Samples and Potential Triggers

**Figure 4:**
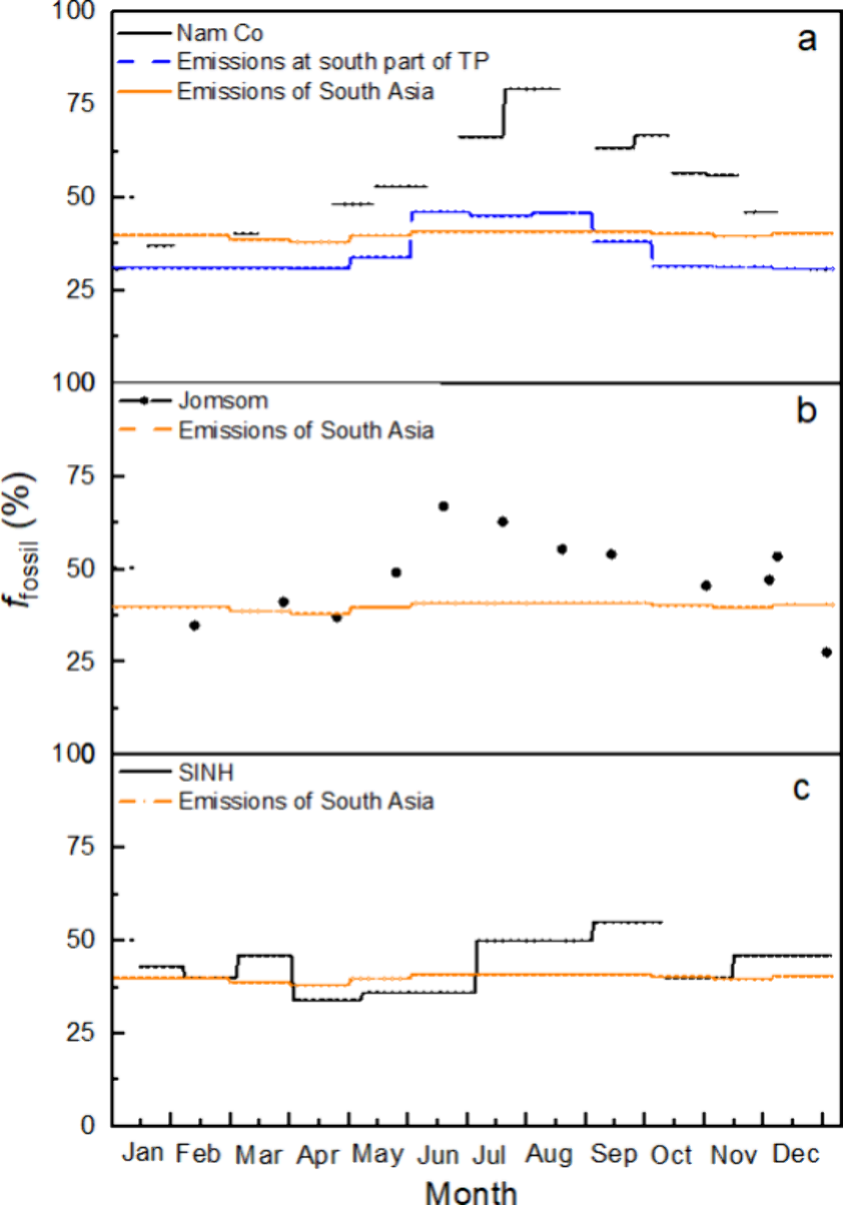
Seasonal variations of *f*_fossil_ BC at Nam Co station (a), Jomsom (b),^25^ and SINH (c).^26^ Note: *f*_fossil_ BC of the south part of the TP and South Asia was calculated from the PKU emission inventories (Table. S3).

Comparisons of f_fossil_ BC values among this study, Jomsom, SINH and emission inventories are presented in Figure 4. It should be highlighted that there are three potential sources of uncertainties when these comparisons: First, aerosol samples collected in this study, as well as those from Jomsom and SINH, reflect localized site characteristics, whereas emission inventories represent broader regional averages. Second, the sampling periods for these study sites differ from the temporal scope of the emission inventories. Third, differences in the residence times of BC from various sources can lead to discrepancies between source-region inventories and the measurements at receptor sites. Despite these uncertainties, the comparisons provide valuable insights into source contributions and the consistency of *f*_fossil_ BC estimates across different approaches.

Distinct seasonal variations in *f*_fossil_ BC were observed in PM_2.5_ samples at the Nam Co station. During the monsoon season, *f*_fossil_ BC in PM_2.5_ samples at Nam Co station were significantly higher than those recorded in SINH, Jomsom and Nam Co station itself during the nonmonsoon season (Figure 4). Meanwhile, *f*_fossil_ BC at Nam Co station during the monsoon season was higher than estimates from emission inventories for both South Asia and the TP (Figure 4a). This suggests that fractionation between BC_bb_ and BC_ff_ during wet deposition plays a crucial role in the observed seasonal trends, as further supported by the clear difference between *f*_fossil_ BC in precipitation and PM_2.5_ samples (Figure 3). Consequently, it is proposed that more BC_bb_ than BC_ff_ is preferentially removed from the atmosphere during precipitation events, resulting in a peak in *f*_fossil_ BC at the Nam Co station during the monsoon season. BC_ff_, being more hydrophobic and smaller in particle size than that of BC_bb_, is more likely to be transported over long distance.^21^ Meanwhile, BC_bb_ tends to be surrounded by a higher proportion of other components, such as brown carbon, compared to BC_ff_, making BC_bb_ heavier than the BC_ff_ aerosol. This could also cause fractionation between these two types of BC through gravitational settling over long distances.^36^ In fact, a similar phenomenon was also observed at Jomsom and SINH. While local sources, such as vehicles and cooking emissions, may contribute to the aerosols collected at these two sites,^25^ increased precipitation events during monsoon season also play an important role in the elevated f_fossil_ BC during this period (Figure 4). This assumption is also supported by the significant relationship between *f*_fossil_ BC in PM_2.5_ and precipitation amount during the collection period (Figure S3), similar to that reported for *f*_fossil_ of WIPC in PM_2.5_ samples collected at Nam Co station,^23^ indicating a consistent wet deposition mechanism for both WIPC and BC. Additionally, lower active fire spots during the monsoon season (Figure S4c) is also consistent with slightly lower BC_bb_ (and commensurately higher *f*_fossil_ BC) in this period.

IGP is generally considered the primary source region for BC aerosol in the TP.^37^ However, the seasonal variation of *f*_fossil_ BC at SINH was different with that of Nam Co station (Figure 4). While the *f*_fossil_ BC values at both sites were similar during the nonmonsoon season, the corresponding values at Nam Co station during the monsoon season were significantly higher than those at SINH (Figure 4).^26^ For instance, the ratio of *f*_fossil_ BC between the monsoon season and nonmonsoon seasons at SINH is 1.22, lower than the ratio of 1.47 observed at Nam Co station. Jomsom showed an intermediate value of 1.42, indicating an increasing seasonal variation trend of *f*_fossil_ BC from the background sites of South Asia to the remote Himalayas and the inner TP. This may be caused by the cumulative impact caused by multiple precipitation events that BC undergoes as it is transported from source regions to remote sites. This refers to the fact that BC emitted from IGP can be transported over long distances and undergo multiple precipitation deposition events before reaching the Nam Co station. In other words, BC collected at the Nam Co station is subjected to longer transport distances and more frequent precipitation events compared to BC collected at SINH, which is closer to the emission sources. This is further supported by comparing the seasonal variations in *f*_fossil_ BC with those from the emission inventories^38^ (Figure 4). First, *f*_fossil_ BC estimates in South Asia and the TP based on emission inventories showed little seasonal variation, which is consistent with the *f*_fossil_ BC at three sites during the nonmonsoon season, as all regions are dominated by biomass burning emissions. Second, the difference between the *f*_fossil_ BC from emission inventories and observed values was smaller during the nonmonsoon season than that during the monsoon season at all three sites. Because *f*_fossil_ BC at the Nam Co station during the monsoon season was significantly higher than the estimates from both the TP and South Asia emission inventories (Figure 4a), the most likely explanation is the fractionation of BC_ff_ and BC_bb_ due to increased precipitation events experienced during transportation. Therefore, BC_ff_ has a much longer atmospheric lifetime than that of BC_bb_ in the wet season. Based on *f*_fossil_ BC in precipitation and aerosols, it is estimated that the scavenging ratio of BC_bb_ was around 4.47 times higher than that of BC_ff_ during the monsoon season at Nam Co station. Despite the fact that the scavenging ratio of BC_bb_ and BC_ff_ likely varies spatially, the atmospheric lifetime of BC_ff_ should be longer than that of BC_bb_ at both source and receptor regions during the wet season.

In conclusion, heavy precipitation is the primary factor driving high *f*_fossil_ BC in PM_2.5_ at the Nam Co station during the monsoon season. Due to its longer transport potential, BC_ff_ is more likely to reach remote regions during the monsoon season with frequent precipitation. Additionally, *f*_fossil_ BC in PM_2.5_ samples collected at Nam Co station in January and March were comparable to or even lower than levels in South Asia (Figure 4a), likely reflecting contributions from biomass combustion emissions within the TP itself (Figure S4). For example, seasonal variation of *f*_fossil_ BC at the Nam Co station more closely resembles that of emissions from the TP than from South Asia (Figure 4a). Typically, the hydrophilicity of aged BC increases compared to freshly emitted aerosols as it becomes coated or internally mixed with non-BC components during atmospheric transport.^39^ This enhanced hydrophilicity likely contributes to the observed lower contribution of IGP-sourced BC during monsoon season compared to the nonmonsoon season at the study site. Despite this, the *f*_fossil_ BC at Nam Co station and the other two sites (Figure 4) showed a significant influence from IGP emissions during the non–monsoon season, mainly due to sparse precipitation and prevailing winds from South Asia (Figure S4). Similar conclusions were also reached from studies on WIPC in PM_2.5_ samples at Nam Co station^23^ and the aerosol transportation model.^40^ Another study proposed that decreased precipitation greatly increased transport efficiency of BC from South Asia to the Himalayas,^41^ further supporting this assumption.

### 3.3 Implication

In this study, we comprehensively investigated *f*_fossil_ BC in PM_2.5_ samples collected from a remote site in the inner TP. We found that *f*_fossil_ BC in precipitation samples was significantly lower than that in PM_2.5_ samples, due to the fractionation between BC_bb_ and BC_ff_ caused by precipitation. Specifically, during the rainy monsoon season, *f*_fossil_ BC in PM_2.5_ samples reached as high as 80.35%, much higher than that observed and estimated from emission inventories in potential source regions (i.e., IGP). These results suggest that BC transported from South Asia to TP undergoes extensive wet deposition during the monsoon season, leading to a relative enrichment of *f*_fossil_ BC in the atmosphere. In contrast, due to sparse precipitation during the dry nonmonsoon season, BC emitted from South Asia can be effectively transported to the inner TP.^42^ This study provides strong geochemical evidence that cross-boundary transport of BC from heavily polluted South Asia to the remote inner TP occurs year-round, with much higher efficiency during the non–monsoon season. Normally, wet deposition accounts for a large fraction of the total deposition of BC.^43^ In this study, we found that the marked seasonal fluctuations of *f*_fossil_ BC in PM_2.5_ samples at this remote site are mainly influenced by precipitation patterns rather than changes in source regions.

Emission inventories are crucial for studying climate forcing and the transport of BC in the atmosphere.^30^ However, this study shows that the seasonal variation of *f*_fossil_ BC in the atmosphere at the Nam Co station differs more significantly from both emission inventories and observations at background sites in South Asia. Because BC_bb_ is removed from the atmosphere more efficiently than BC_ff_ during transport from source regions to remote sites, the significance of BC_bb_ in climate forcing in remote regions is likely lower than suggested by emission inventories. Consequently, BC_bb_ and BC_ff_ should be treated separately in atmospheric transport models to improve the accuracy of BC fate modeling and their influence on climate forcing.

## Supporting Information Available

Data of aerosol samples of study site; data of precipitation samples of study site; data of BC from emission inventories; location of study site; *f*_fossil_ BC between PM_2.5_ samples collected at study site and its North bank; relationship between *f*_fossil_ BC in PM_2.5_ samples and precipitation amount; five-day backward air mass trajectories in different seasons at study site (PDF)

## References

[ref1] Bond T. C.; Doherty S. J.; Fahey D. W.; Forster P. M.; Berntsen T.; DeAngelo B. J.; Flanner M. G.; Ghan S.; Kärcher B.; Koch D. Bounding the role of black carbon in the climate system: A scientific assessment. Journal of Geophysical Research: Atmospheres 2013, 118, 1840 10.1002/jgrd.50176.

[ref2] Kang S.; Zhang Y.; Qian Y.; Wang H. A review of black carbon in snow and ice and its impact on the cryosphere. Earth-Science Reviews 2020, 210, 103346 10.1016/j.earscirev.2020.103346.

[ref3] Flanner M. G.; Zender C. S.; Randerson J. T.; Rasch P. J. Present-day climate forcing and response from black carbon in snow. Journal of Geophysical Research: Atmospheres 2007, 112 (D11), D11202 10.1029/2006JD008003.

[ref4] Yang J.; Kang S.; Chen D.; Zhao L.; Ji Z.; Duan K.; Deng H.; Tripathee L.; Du W.; Rai M.; et al. South Asian black carbon is threatening the water sustainability of the Asian Water Tower. J. N. C 2022, 13 (1), 7360 10.1038/s41467-022-35128-1. PMC9712424 36450769

[ref5] Li C.; Bosch C.; Kang S.; Andersson A.; Chen P.; Zhang Q.; Cong Z.; Chen B.; Qin D.; Gustafsson Ö. Sources of black carbon to the Himalayan–Tibetan Plateau glaciers. Nat. Commun. 2016, 7, 12574 10.1038/ncomms12574. 27552223 PMC4996979

[ref6] Lu Z.; Streets D. G.; Zhang Q.; Wang S. A novel back-trajectory analysis of the origin of black carbon transported to the Himalayas and Tibetan Plateau during 1996–2010. Geophys. Res. Lett. 2012, 39 (1), L01809 10.1029/2011GL049903.

[ref7] Hu T.; Cao J.; Zhu C.; Zhao Z.; Liu S.; Zhang D. Morphologies and elemental compositions of local biomass burning particles at urban and glacier sites in southeastern Tibetan Plateau: Results from an expedition in 2010. Sci. Total Environ. 2018, 628, 772–781. 10.1016/j.scitotenv.2018.02.073. 29454217

[ref8] Kopacz M.; Mauzerall D. L.; Wang J.; Leibensperger E. M.; Henze D. K.; Singh K. Origin and radiative forcing of black carbon transported to the Himalayas and Tibetan Plateau. Atmospheric Chemistry and Physics 2011, 11 (6), 2837–2852. 10.5194/acp-11-2837-2011.

[ref9] Li C.; Kang S.; Yan F. Importance of Local Black Carbon Emissions to the Fate of Glaciers of the Third Pole. Environ. Sci. Technol. 2018, 52, 14027–14028. 10.1021/acs.est.8b06285. 30499660

[ref10] Gustafsson Ö.; Kruså M.; Zencak Z.; Sheesley R. J.; Granat L.; Engström E.; Praveen P. S.; Rao P. S. P.; Leck C.; Rodhe H. Brown clouds over South Asia: biomass or fossil fuel combustion?. Science 2009, 323 (5913), 495–498. 10.1126/science.1164857. 19164746

[ref11] Szidat S.; Jenk T. M.; Synal H.-A.; Kalberer M.; Wacker L.; Hajdas I.; Kasper-Giebl A.; Baltensperger U. Contributions of fossil fuel, biomass-burning, and biogenic emissions to carbonaceous aerosols in Zurich as traced by 14C. Journal of Geophysical Research: Atmospheres (1984–2012) 2006, 111 (D7), D07206 10.1029/2005JD006590.

[ref12] Chen B.; Andersson A.; Lee M.; Kirillova E. N.; Xiao Q.; Krusa M.; Shi M.; Hu K.; Lu Z.; Streets D. G.; et al. Source Forensics of Black Carbon Aerosols from China. Environ. Sci. Technol. 2013, 47 (16), 9102–9108. 10.1021/es401599r. 23844635

[ref13] Mouteva G. O.; Randerson J. T.; Fahrni S. M.; Bush S. E.; Ehleringer J. R.; Xu X.; Santos G. M.; Kuprov R.; Schichtel B. A.; Czimczik C. I. Using radiocarbon to constrain black and organic carbon aerosol sources in Salt Lake City. Journal of Geophysical Research: Atmospheres 2017, 122 (18), 9843–9857. 10.1002/2017JD026519.

[ref14] Winiger P.; Andersson A.; Eckhardt S.; Stohl A.; Gustafsson O. The sources of atmospheric black carbon at a European gateway to the Arctic. Nat. Commun. 2016, 7, 12776 10.1038/ncomms12776. 27627859 PMC5027618

[ref15] Winiger P.; Andersson A.; Eckhardt S.; Stohl A.; Semiletov I. P.; Dudarev O. V.; Charkin A.; Shakhova N.; Klimont Z.; Heyes C.; et al. Siberian Arctic black carbon sources constrained by model and observation. Proc. Natl. Acad. Sci. U.S.A. 2017, 114 (7), E1054–E1061. 10.1073/pnas.1613401114. 28137854 PMC5320976

[ref16] Ni H.; Yao P.; Zhu C.; Qu Y.; Tian J.; Ma Y.; Yang L.; Zhong H.; Huang R. J.; Dusek U. Non-Fossil Origin Explains the Large Seasonal Variation of Highly Processed Organic Aerosol in the Northeastern Tibetan Plateau (3,200 m asl). Geophys. Res. Lett. 2023, 50 (13), e2023GL104710 10.1029/2023GL104710.

[ref17] Li C.; Yan F.; Kang S.; Yan C.; Hu Z.; Chen P.; Gao S.; Zhang C.; He C.; Kaspari S.; et al. Carbonaceous matter in the atmosphere and glaciers of the Himalayas and the Tibetan plateau: An investigative review. Environ. Int. 2021, 146, 106281 10.1016/j.envint.2020.106281. 33395932

[ref18] Cong Z.; Kang S.; Smirnov A.; Holben B. Aerosol optical properties at Nam Co, a remote site in central Tibetan Plateau. Atmospheric Research 2009, 92 (1), 42–48. 10.1016/j.atmosres.2008.08.005.

[ref19] Li C.; Yan F.; Kang S.; Chen P.; Han X.; Hu Z.; Zhang G.; Hong Y.; Gao S.; Qu B.; et al. Re-evaluating black carbon in the Himalayas and the Tibetan Plateau: concentrations and deposition. Atmospheric Chemistry and Physics 2017, 17 (19), 11899–11912. 10.5194/acp-17-11899-2017.

[ref20] Ming J.; Xiao C.; Sun J.; Kang S.; Bonasoni P. Carbonaceous particles in the atmosphere and precipitation of the Nam Co region, central Tibet. Journal of Environmental Sciences 2010, 22 (11), 1748–1756. 10.1016/S1001-0742(09)60315-6. 21235163

[ref21] Pio C. A.; Legrand M.; Oliveira T.; Afonso J.; Santos C.; Caseiro A.; Fialho P.; Barata F.; Puxbaum H.; Sanchez-Ochoa A. Climatology of aerosol composition (organic versus inorganic) at nonurban sites on a west-east transect across Europe. Journal of Geophysical Research-Atmospheres 2007, 112 (D23), D23S02 10.1029/2006JD008038.

[ref22] Wang J.; Wang J.; Cai R.; Liu C.; Jiang J.; Nie W.; Wang J.; Moteki N.; Zaveri R. A.; Huang X. Unified theoretical framework for black carbon mixing state allows greater accuracy of climate effect estimation. Nat. Commun. 2023, 14 (1), 2703 10.1038/s41467-023-38330-x. 37164951 PMC10172310

[ref23] Li C.; Zhang C.; Kang S.; Xu Y.; Yan F.; Liu Y.; Rai M.; Zhang H.; Chen P.; Wang P.; et al. Weak transport of atmospheric water-insoluble particulate carbon from South Asia to the inner Tibetan Plateau in the monsoon season. Science of The Total Environment 2024, 922, 171321 10.1016/j.scitotenv.2024.171321. 38423306

[ref24] Rodríguez B.; Huang L.; Santos G.; Zhang W.; Vetro V.; Xu X.; Kim S.; Czimczik C. Seasonal cycle of isotope-based source apportionment of elemental carbon in airborne particulate matter and snow at Alert, Canada. J. J. o. G. R. A 2020, 125 (23), e2020JD033125 10.1029/2020JD033125.

[ref25] Chen P.; Kang S.; Li C.; Hu Z.; Tripathee L.; Rai M.; Pu T.; Yin X.; Gustafsson Ö. Carbonaceous aerosol transport from the Indo-Gangetic Plain to the Himalayas: Carbon isotope evidence and light absorption characteristics. J. G. F. 2023, 14 (2), 101516 10.1016/j.gsf.2022.101516.

[ref26] Budhavant K.; Andersson A.; Bosch C.; Kruså M.; Kirillova E. N.; Sheesley R. J.; Safai P. D.; Rao P. S. P.; Gustafsson Ö. Radiocarbon-based source apportionment of elemental carbon aerosols at two South Asian receptor observatories over a full annual cycle. Environmental Research Letters 2015, 10 (6), 064004 10.1088/1748-9326/10/6/064004.

[ref27] Dasari S.; Andersson A.; Stohl A.; Evangeliou N.; Bikkina S.; Holmstrand H.; Budhavant K.; Salam A.; Gustafsson O. r. Source quantification of South Asian black carbon aerosols with isotopes and modeling. Environ. Sci. Technol. 2020, 54 (19), 11771–11779. 10.1021/acs.est.0c02193. 32885963 PMC7586323

[ref28] Winiger P.; Barrett T.; Sheesley R.; Huang L.; Sharma S.; Barrie L. A.; Yttri K. E.; Evangeliou N.; Eckhardt S.; Stohl A.; et al. Source apportionment of circum-Arctic atmospheric black carbon from isotopes and modeling. Science advances 2019, 5 (2), eaau8052 10.1126/sciadv.aau8052. 30788434 PMC6374108

[ref29] Yan F.; He C.; Kang S.; Chen P.; Hu Z.; Han X.; Gautam S.; Yan C.; Zheng M.; Sillanpää M. Deposition of organic and black carbon: direct measurements at three remote stations in the Himalayas and Tibetan Plateau. Journal of Geophysical Research: Atmospheres 2019, 124, 9702 10.1029/2019JD031018.

[ref30] Bond T. C.; Doherty S. J.; Fahey D. W.; Forster P. M.; Berntsen T.; DeAngelo B. J.; Flanner M. G.; Ghan S.; Kaercher B.; Koch D.; et al. Bounding the role of black carbon in the climate system: A scientific assessment. Journal of Geophysical Research-Atmospheres 2013, 118 (11), 5380–5552. 10.1002/jgrd.50171.

[ref31] Andersson A.; Deng J.; Du K.; Zheng M.; Yan C.; Skold M.; Gustafsson O. Regionally-Varying Combustion Sources of the January 2013 Severe Haze Events over Eastern China. Environ. Sci. Technol. 2015, 49 (4), 2038–2043. 10.1021/es503855e. 25569822

[ref32] Szidat S.; Ruff M.; Perron N.; Wacker L.; Synal H. A.; Hallquist M.; Shannigrahi A. S.; Yttri K. E.; Dye C.; Simpson D. Fossil and non-fossil sources of organic carbon (OC) and elemental carbon (EC) in Goteborg, Sweden. Atmospheric Chemistry and Physics 2009, 9 (5), 1521–1535. 10.5194/acp-9-1521-2009.

[ref33] Jenk T. M.; Szidat S.; Schwikowski M.; Gggeler H. W.; Brutsch S.; Wacker L.; Synal H. A.; Saurer M. Radiocarbon analysis in an Alpine ice core: record of anthropogenic and biogenic contributions to carbonaceous aerosols in the past (1650–1940). Atmospheric Chemistry and Physics 2006, 6 (12), 5381–5390. 10.5194/acp-6-5381-2006.

[ref34] Zhang X.; Ming J.; Li Z.; Wang F.; Zhang G. The online measured black carbon aerosol and source orientations in the Nam Co region, Tibet. Environmental science and pollution research international 2017, 24, 25021 10.1007/s11356-017-0165-1. 28920168 PMC5683056

[ref35] Zhang Y. L.; Cerqueira M.; Salazar G.; Zotter P.; Hueglin C.; Zellweger C.; Pio C.; Prevot A. S. H.; Szidat S. Wet deposition of fossil and non-fossil derived particulate carbon: Insights from radiocarbon measurement. Atmos. Environ. 2015, 115, 257–262. 10.1016/j.atmosenv.2015.06.005.

[ref36] Pósfai M.; Gelencsér A.; Simonics R.; Arató K.; Li J.; Hobbs P. V.; Buseck P. R. Atmospheric tar balls: Particles from biomass and biofuel burning. Journal of Geophysical Research: Atmospheres 2004, 109 (D6), D06213 10.1029/2003JD004169.

[ref37] Yang J.; Kang S.; Ji Z. Critical contribution of south Asian residential emissions to atmospheric black carbon over the Tibetan plateau. Science of The Total Environment 2020, 709, 135923 10.1016/j.scitotenv.2019.135923. 31884284

[ref38] Xu H.; Ren Y. a.; Zhang W.; Meng W.; Yun X.; Yu X.; Li J.; Zhang Y.; Shen G.; Ma J. J. E. S.; et al. Updated global black carbon emissions from 1960 to 2017: improvements, trends, and drivers. Environ. Sci. Technol. 2021, 55 (12), 7869–7879. 10.1021/acs.est.1c03117. 34096723

[ref39] Vignati E.; Karl M.; Krol M.; Wilson J.; Stier P.; Cavalli F. Sources of uncertainties in modelling black carbon at the global scale. Atmospheric chemistry and physics 2010, 10 (6), 2595–2611. 10.5194/acp-10-2595-2010.

[ref40] Hu Y.; Yu H.; Kang S.; Yang J.; Chen X.; Yin X.; Chen P. Modeling the transport of PM10, PM2. 5, and O3 from South Asia to the Tibetan Plateau. J. A. R. 2024, 303, 107323 10.1016/j.atmosres.2024.107323.

[ref41] Li C.; Yan F.; Zhang C.; Kang S.; Rai M.; Zhang H.; Hu S.; He C. Coupling of decreased snow accumulation and increased light-absorbing particles accelerates glacier retreat in the Tibetan Plateau. Science of The Total Environment 2022, 809, 151095 10.1016/j.scitotenv.2021.151095. 34688751

[ref42] Kaspari S. D.; Schwikowski M.; Gysel M.; Flanner M. G.; Kang S.; Hou S.; Mayewski P. A. Recent increase in black carbon concentrations from a Mt. Everest ice core spanning 1860–2000 AD. Geophys. Res. Lett. 2011, 38, L04703 10.1029/2010GL046096.

[ref43] Browse J.; Carslaw K. S.; Arnold S. R.; Pringle K.; Boucher O. The scavenging processes controlling the seasonal cycle in Arctic sulphate and black carbon aerosol. Atmospheric Chemistry and Physics 2012, 12 (15), 6775–6798. 10.5194/acp-12-6775-2012.

