# Supplementary material for: Longer Lifetime of BC from Fossil Fuel Combustion than from Biomass Burning: Δ14C Evidence
Source: Environ Sci Technol. 2025 Feb 25;59(9):4571–7. doi: 10.1021/acs.est.4c10040 (PMC11912310; doi:10.1021/acs.est.4c10040)
Supplement: Supplementary file 1 — es4c10040_si_001.pdf [file es4c10040_si_001.pdf]

Supplementary Information for

Longer lifetime of BC from fossil fuel combustion than from biomass burning: $\Delta^{14}C$ evidence

Chaoliu Li[a,e], Zhaofu Hu[a], Shichang Kang[a,d*], Elena N. Kirillova[b,f], Fangping Yan[a, b], Pengfei Chen[a], Guofeng Shen[c], Thompson T. Jake[e], Örjan Gustafsson[b*]

[a] Key Laboratory of Cryospheric Science and Frozen Soil Engineering, Northwest Institute of Eco-Environment and Resources, Chinese Academy of Sciences, Lanzhou 730000, China;

[b] Department of Environmental Science and the Bolin Centre for Climate Research, Stockholm University, Stockholm 10691, Sweden;

[c]College of Urban and Environmental Sciences, Peking University, Beijing, 100871, China;

[d] University of Chinese Academy of Sciences, Beijing 100049, China;

[e] Department of Earth and Planetary Sciences, Yale University, New Haven, CT 06511, USA;

[f] Institute of Medicine, Ecology and Physical Education, Ulyanovsk State University, Ulyanovsk, Russian Federation, 432017

[*]Corresponding Authors: Shichang Kang (shichang.kang@lzb.ac.cn; +86-931-4967368), Örjan Gustafsson (orjan.gustafsson@aces.su.se; +46-70-3247317)

Summary: 10 pages, 3 tables, and 4 figures.

Table S1. Concentrations, isotopic ($\Delta^{14}C$ and $\delta^{13}C$) compositions and fossil fuel contributions of BC in the aerosol samples of Nam Co station.

Collecting date	Season	OC(ng m^{-3})	BC(ng m^{-3})	$\delta^{13}C$ (‰)	$\Delta^{14}C$(‰)	f_{fossil} (%)
2016/12/6	N	623.9	108.2	-	-363.5	40.51
2017/1/18	N	420.74	70.37		-327.44	37.14
2017/3/3	N	802.23	100.51	-	-361.2	40.3
2017/4/22	N	1068.72	166.92	-25.1	-446.5	48.27
2017/5/14	N	380.72	113.26	-25.3	-497.1	53
2017/6/25	M	312.62	41.69	-25.6	-639.9	66.35
2017/7/17	M	278.22	52.67	-26.1	-777.8	79.23
2017/9/2	M	414.03	63.35	-	-608.2	63.38
2017/9/21	M	504.78	75.42	-	-644.9	66.81
2017/10/11	N	726.14	109.77	-	-535.8	56.62
2017/10/28	N	666.85	105.61	-	-529.8	56.06
2017/11/17	N	332.57	48.76	-	-423.7	46.14
2017/12/20	N	1332.09	147.18	-24.4	-260	30.84
2018/1/8	N	1256.69	42.52	-	-198.49	25.09
2018/2/9	N	595.2	60.21	-	-283.8	33.07
2018/3/1	N	823.91	75.13	-	-275.86	32.32
2018/3/17	N	998.12	81.56	-	-222.3	27.32
2018/4/1	N	766.23	57.9	-	-354.23	39.65
2018/4/16	N	691.26	66.23	-	-	-
2018/4/20	N	725.68	98.05	-23.8	-321.49	36.59
2018/5/1	N	475.98	65.4	-	-457.39	49.29
2018/5/17	N	537.81	123.6	-	-480.21	51.42
2018/6/5	M	252.79	29.35	-26.3	-575.44	60.32
2018/6/18	M	143.11	61.13	-	-	-
2018/7/20	M	309.68	43.5	-	-648.9	67.19
2018/8/7	M	407.81	56.24	-	-789.7	80.35
2018/9/22	M	302.81	57.82	-25.5	-649.83	67.27
2019/2/22	N	434.59	32.66	-25.8	-343.32	38.63
2019/3/10	N	542.46	42.15	-	-	-
2019/3/25	N	472.97	47.38	-23.3	-400.85	44
2019/4/9	N	519.03	49.9	-21.5	-475.25	50.96
2019/5/2	N	907.29	97.25	-23.5	-413.75	45.21
2019/5/30	M	659.4	51.15	-24.9	-599.24	62.55
2018/6/29	M-NB	321.34	53.7	-	-729.2	74.69
2018/7/30	M-NB	362.21	37.41	-27.6	-770.9	78.59
2019/4/4	N-NB	451.14	56.74	-23	-458.38	49.38
2019/5/5	N-NB	822.45	74.18	-25.5	-402.83	44.19
2019/5/30	M-NB	539.8	63.15	-24.8	-570.48	59.86
2019/7/2	M-NB	1313.15	52.7	-24.9	-362.16	40.39
Average-monsoon		358.53	53.23	-25.68	-659.32	68.16
SD-monsoon		144.94	12.87	0.55	75.07	7.02
Average-non-monsoon		602.47	71.30	-24.83	-477.77	51.19
SD-non-monsoon		295.49	31.50	1.41	161.75	15.12

Note: N, M and -NB mean non-monsoon, monsoon periods and those samples collected at north bank, respectively.

Table S2. $\Delta^{14}C$ and $\delta^{13}C$ compositions and fossil fuel contributions of BC in precipitation samples at Nam Co station.

Precipitation event	$\Delta^{14}C$ (‰)	Fossil contribution (%)	$\delta^{13}C$ (‰)
2018/8/2, 10/5	-397.9	43.7	-24.8
2019/7/4, 7/17	-203.4	25.6	-24.4
2019/7/6, 7/11, 7/15, 7/16, 7/17, 7/22	-258.0	30.7	-24.3
2019/7/23, 8/1, 8/3, 8/4	-479.2	51.3	-25.4
2019/8/4, 8/5, 8/6, 8/24	-100.3	15.9	-26.7

Table S3. BC emission (Kt) and f_{fossil} BC (%) of south part of the TP (A, C) and South Asia (B, D) estimated from emission inventories (https://gems.pku.edu.cn).

	Jan	Feb	Mar	Apr	May	Jun	Jul	Aug	Sep	Oct	Nov	Dec
A	0.31	0.28	0.31	0.30	0.28	0.20	0.21	0.20	0.24	0.30	0.30	0.31
B	24.66	23.06	25.39	24.99	24.70	23.26	24.03	24.03	23.27	24.36	23.95	24.35
C	31.22	31.14	31.14	31.05	33.96	46.21	45.18	45.90	38.20	31.65	31.29	30.86
D	39.98	39.97	38.73	38.06	39.79	40.88	40.89	40.89	40.86	40.35	39.74	40.43

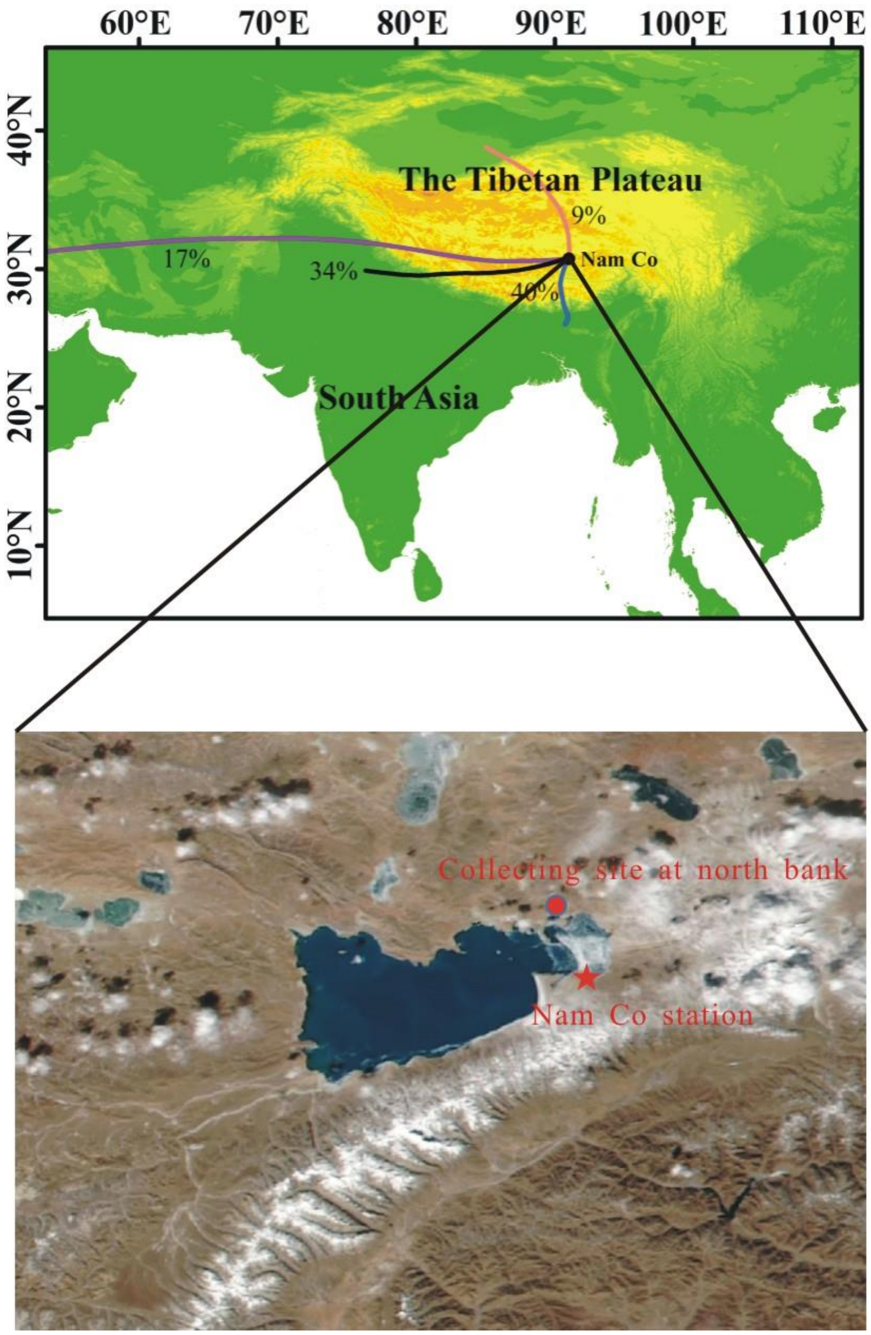

Figure S1. Collecting site at north bank of Nam Co Lake relative to that of Nam Co station with the five-day backward air mass trajectories by HYSPLIT model analysis for one year during study period. The percentage value means the frequency of air masses coming from a certain direction. Note: $PM_{2.5}$ samples collected only during monsoon period at north bank to check the potential local emissions to BC collected at Nam Co station.

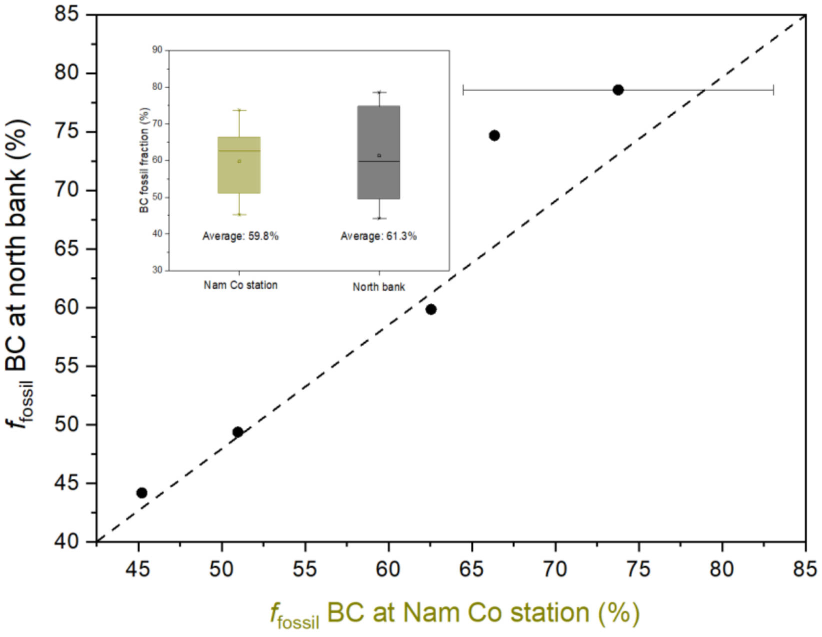

Figure S2. Comparison f_{fossil} BC between $PM_{2.5}$ samples collected at Nam Co station and North bank of Nam Co.

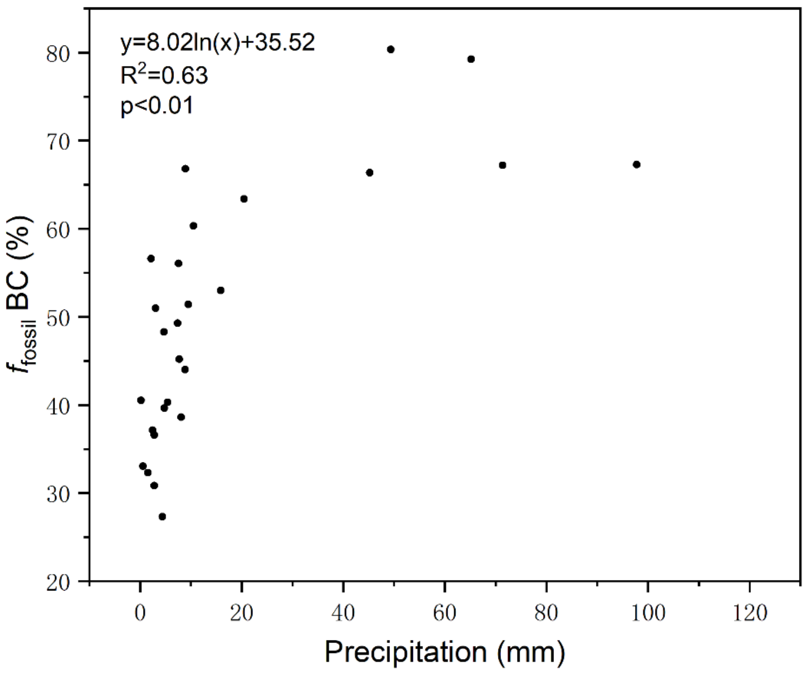

Figure S3. Significant relationship between f_{fossil} BC in $PM_{2.5}$ samples and precipitation amount during sample collection period.

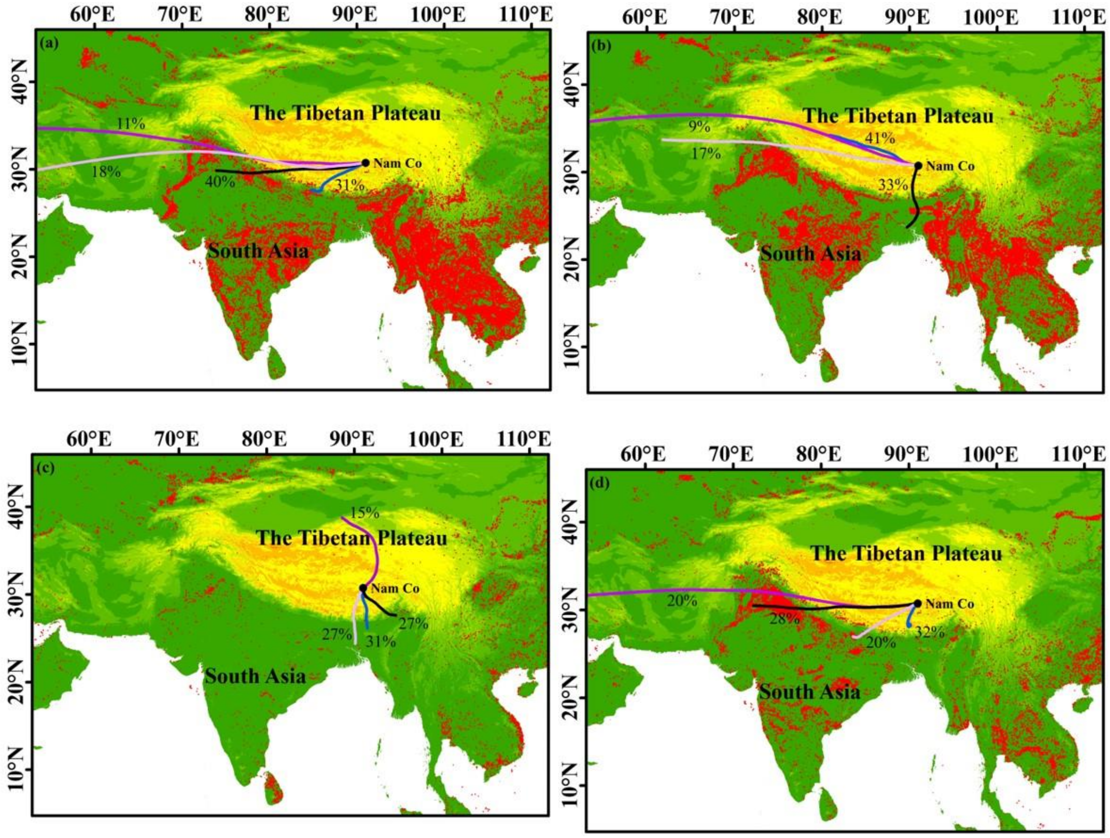

Figure S4. The five-day backward air mass trajectories by HYSPLIT model analysis in different seasons (a: Winter, b: Pre-monsoon, c: Monsoon, d: Post-monsoon) during sampling period at Nam Co station. The background map is spatial distribution of active fire spots (read points) (https://firms.modaps.eosdis.nasa.gov/firemap/) [1].

Supplementary References

(1) Li, Y.; Yan, F.; Kang, S.; Zhang, C.; Chen, P.; Hu, Z.; Li, C. Sources and light absorption characteristics of water-soluble organic carbon (WSOC) of atmospheric particles at a remote area in inner Himalayas and Tibetan Plateau. *Atmospheric Research* **2021**, *253*, 105472.
